# A Practical Essay on Stricture of the Rectum

**Published:** 1833-10

**Authors:** 


*A Practical Essay on Stricture of the Rectum: illustrated by Cases, shewing the Connexion of that Disease with Prolapsus of the Bowel, Piles, Fistula, Affections of the Urinary Organs and of the Womb, &c.* By Frederick Salmon, F.R.C.S. *Fourth Edition.* London. 8vo. pp. 317.

This work is worth consulting chiefly as a repository of rare cases, of which it contains a considerable number. The following is an abstract of one of those added in the present edition, and is certainly a remarkable one.

Mrs. B., a lady, aged fifty-nine, had long suffered from diarrhœa, with occasionally bloody dejections, and had experienced a violent attack of inflammation of the bowels and retention of urine; and, about nine months before she sought the advice of Mr. Salmon, she thought she perceived fæces in her urine. Our author first saw her in March 1831, when he found her "suffering from pain in the region of the sigmoid flexure of the colon and the bladder, extending into the rectum and vagina. She had a frequent inclination to make water, and her bowels were relieved, upon an average, ten times in every twelve hours: she complained that, at the period of passing her stools, the scalding and pain in the fundament almost distracted her; she also suffered much uneasiness in the front passage, particularly when she began to pass her motion, but, as her bowels were emptied, this generally subsided. The stools did not always come the wrong way, but appeared to depend on the looseness of her bowels; the quantity which passed in this manner increasing or diminishing according to the relaxed or confined state of her body." (P. 137.)

On examination, our author found a stricture at the sigmoid flexure of the colon, but no communication between the rectum and vagina could be detected. The treatment consisted chiefly of opium administered by the mouth and *per anum.* The unfortunate patient lingered on till the 27th of August, in the same year, when she expired. The following were the post-mortem appearances:

"*Sectio Cadaveris.* August 29th, two P.M. Though there was much general emaciation of the body, the fat over the parietes of the abdomen was of considerable thickness. Upon making the usual incision through the abdominal muscles, the peritoneum covering the rectus muscle was found closely adherent to the sigmoid flexure of the colon, the adhesions being firm, and evidently of some standing. The membrane at this part, and in that portion of it which was reflected upon the bladder, and over the lower part of the colon, was thickened in texture, and of a dark-green colour. The descending colon, sigmoid flexure, and that portion of the rectus muscle which was agglutinated to it; the rectum, pelvic viscera, together with some of the soft parts around the anus and meatus externus, were then removed from the body. There was not any disease of the rectum worthy of notice, and, after examining the bowel with the greatest care, no communication could be discovered between it and the vagina. The latter passage laid open, presented no morbid appearance of moment, being only triflingly inflamed. The womb was likewise healthy, as far as its general structure was concerned, but a portion of its cavity towards the fundus was obliterated, from coagulated lymph having become organised in several bands. A very small polypus grew just within the os tincæ. The urethra was next laid open into the bladder; this canal was much dilated and vividly inflamed. The bladder was much contracted, but, notwithstanding the destruction of a part of its fundus, it was not generally diseased, nor was there any material inflammation or thickening of its internal surface; but the colour of this part was of the peculiar green cast before alluded to.

"An incision was now made through a part of the descending colon, its sigmoid flexure, and the first curve of the rectum, so as to bring into view the extent and nature of the stricture. Immediately above the sigmoid flexure, the colon was distended, and contained a considerable quantity of fæces, but at this part the intestine was so contracted, that the point of the fore-finger could hardly be passed through it. The contraction was of a circular form, and about an inch in length, the mucous membrane of the intestine immediately before, throughout, and for some short distance beyond it, being destroyed by ulceration. About three inches of the bowel were adherent by its superior surface to the peritoneum covering the abdominal muscles, while the inferior portion of the intestine was in like manner firmly united to the fundus of the bladder, a part of which was destroyed by ulceration, together with the colon to which it was united, so as to form a direct channel for the passage of the feculent matter. There was not so much thickening of the intestine as might have been expected, and the ulceration appeared as if it had been produced more from acute inflammation, than chronic disease of the part.

"The viscera of the chest and abdomen were healthy." (P. 146.)

